# Classification of crop disease-pest questions based on BERT-BiGRU-CapsNet with attention pooling

**DOI:** 10.3389/fpls.2023.1300580

**Published:** 2023-12-07

**Authors:** Ting Zhang, Dengwu Wang

**Affiliations:** College of Computing, Xijing University, Xi’an, China

**Keywords:** crop disease-pest question, bidirectional gated unit (BiGRU), capsule network (CapsNet), attention pooling, BERT-BiGRU-CapsNet with attention pooling (BBGCAP)

## Abstract

Crop disease-pest question classification is an essential part of pest knowledge intelligent question answering system. A crop disease-pest question classification method is proposed on the basis of bidirectional encoder representations from transformers (BERT), bidirectional gated unit (BiGRU), capsule network (CapsNet), and BERT-BiGRU-CapsNet with attention pooling (BBGCAP). In BBGCAP, the unstructured text data are preprocessed vectorically using BERT, BiGRU is used to extract the deep features of the text, attention pooling is used to assign the corresponding weights to the extracted deep information, and CapsNet is used to route the right alternative. BBGCAP is a synthetic model by integrating the advantages of BERT, BiGRU, CapsNet, and attention pooling. The experimental results on the cucumber-pest question database show that the proposed method is superior to the methods based on traditional template matching, support vector machines (SVM), and convolutional neural network–long short-term memory (LSTM), and the accuracy rates of precision, recall, and F1 are all above 902.15%. This method provides technical support for intelligent question answering system of crop disease-pests.

## 1 Introduction

Crop pest-diseases are one of the important factors that seriously threaten crop yield and quality. Early correct diagnosis and control of pest-diseases can effectively reduce the economic losses caused by pest-diseases (Wang et al., 2022; He et al., 2023). The disease-pests can be effectively prevented and controlled by only timely obtaining crop disease-pest and disease information and taking suitable control measures. Diagnosing and identifying the types of pest-diseases is not an easy task for farmers, especially considering the various pest-diseases and complex environment. The knowledge management of crop disease-pests can provide guidelines for the diagnosis and prevention of pest-diseases, which is a new way to obtain crop pest information in time in precision agriculture (Waheed et al., 2022). However, with the development of the Internet-of-Things technology and the explosive growth of network data, the data related to crop disease-pests also show a highly dispersed, complex, and heterogeneous state, which brings difficulties to farmers, plant-protection experts, and other personnel to quickly and accurately obtain the required information about disease-pests (Cherif, 2022).

It is a key issue to accurately extract useful knowledge such as pathogens, damage sites, and control agents from massive and complex crop pest-related data, where integrating crop disease-pest knowledge is an approach to pest control that aims to maintain harmful insects at tolerable levels, keeping pest populations below the economic damage levels. Bidirectional encoder representations from transformers (BERT) is the first unsupervised, depth bidirectional model for pre-training. It can learn surface features, phrase-level syntactic level features, and semantic level information from a shallow level to a high level, so that the word vector obtained by BERT not only implicitly contains context-level features but also effectively captures sentence-level features (Guo et al., 2021). In recent years, the amount of literature related to pest management has increased rapidly, and a large number of valuable crop pest information is still hidden in unstructured social media, such as the Chinese Agricultural Technology Promotion Q&A community, which adds nearly 10,000 crop pest data every day. Therefore, an effective classification of question sentences is a key technical link in achieving intelligent Q&A by crop producers and managers. At present, in the crop disease-pest knowledge answering system, a lot of question answering systems have been constructed by gradually integrating deep learning–related technologies into the process of practical agricultural production, but there are still some problems (Miguel et al., 2021). (1) There are many types of crop diseases and pests, and relevant knowledge is highly fragmented. (2) Due to the small number of natural language open datasets, short text, sparse features, and difficulty to learn the hidden semantic information in the field of agricultural pests and diseases, it is still difficult to parse and classify pest and disease questions and link attributes from questions to the system. Therefore, it is necessary to conduct the integrated research on pest knowledge and research on Q&A in agricultural pest-disease field to explore a more efficient and accurate Q&A model.

Integrating BERT, bidirectional gated unit (BiGRU), capsule network (CapsNet) and attention pooling, a crop disease-pest question classification method, namely, BBGCAP is proposed. This method has the characteristics of simple structure, fewer training parameters, and fast training speed, which can meet the response time requirements of the question answering system. The main contributions of this paper are summarized as follows:

(1) The unstructured text data are preprocessed vectorically using BERT text pre-training model based on the agricultural domain corpus, and the obtained word vector not only implicitly contains context-level features but also effectively captures sentence-level features.
(2) BiGRU is employed to extract the deep global features of the text, and CapsNet is used to extract the local features of text. CapsNet replaces scalar-output features of convolutional neural network (CNN) with vector-output capsules and pooling layer with dynamic routing algorithm.
(3) Attention pooling is adopted to assign the corresponding weights to the extracted deep information and retain the most significant information at the pooling stage. An intermediate sentence representation generated by BiGRU is used as a reference for local representations produced by the convolutional layer to obtain attention weights. The sentence representation is formed by combining local representations using obtained attention weights.

The rest of this paper is organized as follows. Section 2 simply introduces the related works. BERT-BiGRU-CapsNet with attention pooling, namely, BBGCAP, is described in detail in Section 3. Section 4 shows a preliminary validation analysis of the model in a simulated environment, and, finally, our conclusions and future work are put forward in Section 5.

## 2 Related works

Pest and diseases are two major factors affecting crop yield and quality. Correct detection, diagnosis, and prevention of various crop pest-diseases are the basis of pest-disease management. Farmers have traditionally relied on manual methods to judge and identify pests and diseases, which are time-consuming, expensive, and inaccurate. Traditional crop disease-pest information acquisition methods mainly use keyword-based search engines or shallow semantic analysis, but the returned results are a large number of related websites with vague and redundant answers (El-Ghany et al., 2020; Liu and Wang, 2020).

Deep learning has gained great advantages in crop pest management and has become the standard method for solving most of the technical challenges of crop pest detection, identification, and classification (Liu and Wang, 2021; Lu et al., 2021). Miao et al. (2022) summarized the applications of deep neural networks in pest detection in recent years into three categories, introduced the characteristics and research status of each network, and provided a direction for solving the current problem by describing the methods of multi-information fusion and dataset enhancement. Aiming at the problems of large computational resource and low precision in most CNNs, Zuo et al. (2020) proposed an attention-based lightweight residual network for plant disease recognition. It employs depthwise separable convolution instead of the conventional convolution on the basis of traditional residual neural network. The attention module is introduced to effectively prevent the overfitting problem of the network and enrich local feature learning. To achieve rapid recognition of the common pests in agriculture and forestry, Wang et al. (2020) proposed a pest image recognition method based on deep CNN and compared the performance of different models on Chlamydial Protease-Like Activity Factor (CPAF) dataset, which has 73,635 insect images, including 4,909 original images and 68,726 enhanced images. To enhance the learning ability of micro-lesion features, Chen et al. (2021) selected MobileNet-V2 pre-trained on ImageNet as the backbone network and added the attention mechanism to learn the importance of inter-channel relationship and spatial points for input features. Xin and Wang (2021) proposed a deep convolutional neural network and Google data (DCNN-G) model based on deep learning and fusion of Google data analysis and compared its accuracy with the conventional recognition model. Using CNNs to classify crop pest–disease image quality not only expands the application field of deep learning but also provides a new method for crop pest–disease image quality assessment.

Many deep learning–based entity recognition methods have been presented to identify crop diseases, pests, drug names, and other nouns related to disease-pests. It is a basic part of agricultural knowledge graph, question and answer, and will be implemented as a web application to provide the public with solutions for the prevention and control of crop pest-diseases (Nandhini and Ashokkumar, 2021; Thanammal Indu and Suja Priyadharsini, 2022). The named entities of crop pest-diseases have the common phenomena of complex word formation, word combination, and entity embedding. In particular, in the field of Chinese crop pest-diseases, there are many problems such as multiple entity naming methods, fuzzy entity boundary, inadequate feature extraction, and inconsistent entity boundary labeling. The crop disease-pest–related information is described by complex word-formation and universal phenomena of word combination and entity embedding. To address the above problems, Wang et al. (2022) combined discourse topic and attention mechanism, and proposed the attention-based SoftLexicon with term frequency–inverse document frequency (TF-IDF) for crop disease-pest entity recognition, designed a flow chart to explain the major principles and steps, and explained the model through visual methods. The recognition accuracy of Chinese agricultural pest-diseases was improved by dividing the word sets according to the position of the characters in the word, integrating the discourse theme features into the calculation of lexical information, and introducing the attention mechanism. Guo et al. (2021) used the fine-tuned BERT model to generate context-character–level embedded representations with specific knowledge, introduced adversarial training, and enhanced the generalization and robustness of recognizing rare entities. Miguel et al. (2021) proposed a first step toward a mature, semantically enhanced decision support system for integrated pest management, by collecting data from multiple heterogeneous sources to build a complete agricultural knowledge base and developing a system to help farmers make decisions about pest control. Guo et al. (2020) established an available corpus toward agricultural disease-pests, which contains 11 categories and 34,952 samples, and proposed a Chinese named entity recognition model via joint multi-scale local context features and the self-attention mechanism. The crop disease-pest knowledge Q&A system, as a key module of question answering systems, plays a decisive role in the efficiency of system retrieval. It can answer the questions that farmers encounter during agricultural production and planting, with the core of question sentence classification to match user questions. BiGRU can better understand and deal with dependencies in a language and improve the comprehension of text sequences by considering both historical and future contextual information and using the context information of text to extract the global features of text (Islam et al., 2022). It uses the same parameters to process both forward and backward sequence data, effectively reducing the number of parameters in the model, reducing the risk of overfitting, and improving training and inference efficiency. CapsNet has inherently better generalization capabilities and could theoretically use a considerably smaller number of parameters and get better results (Tao et al., 2022). Attention mechanism can be introduced to assign different weights to BiGRU hidden states through mapping weighting and learning parameter matrix, allocate sufficient attention to key information, highlight the influence of important information, reduce the loss of feature information, and strengthen the influence of important information, so as to improve the accuracy of the model (Meng et al., 2016). As for the complex and diverse semantic information of user questions in agricultural question answering systems, a crop disease-pest question classification method (BBGCAP) is proposed based on BERT, BiGRU, CapsNet, and attention pooling, meeting the needs of users to quickly and accurately obtain the classification results of crop disease questions.

## 3 Classification of crop disease-pest questions

**Figure 1:**
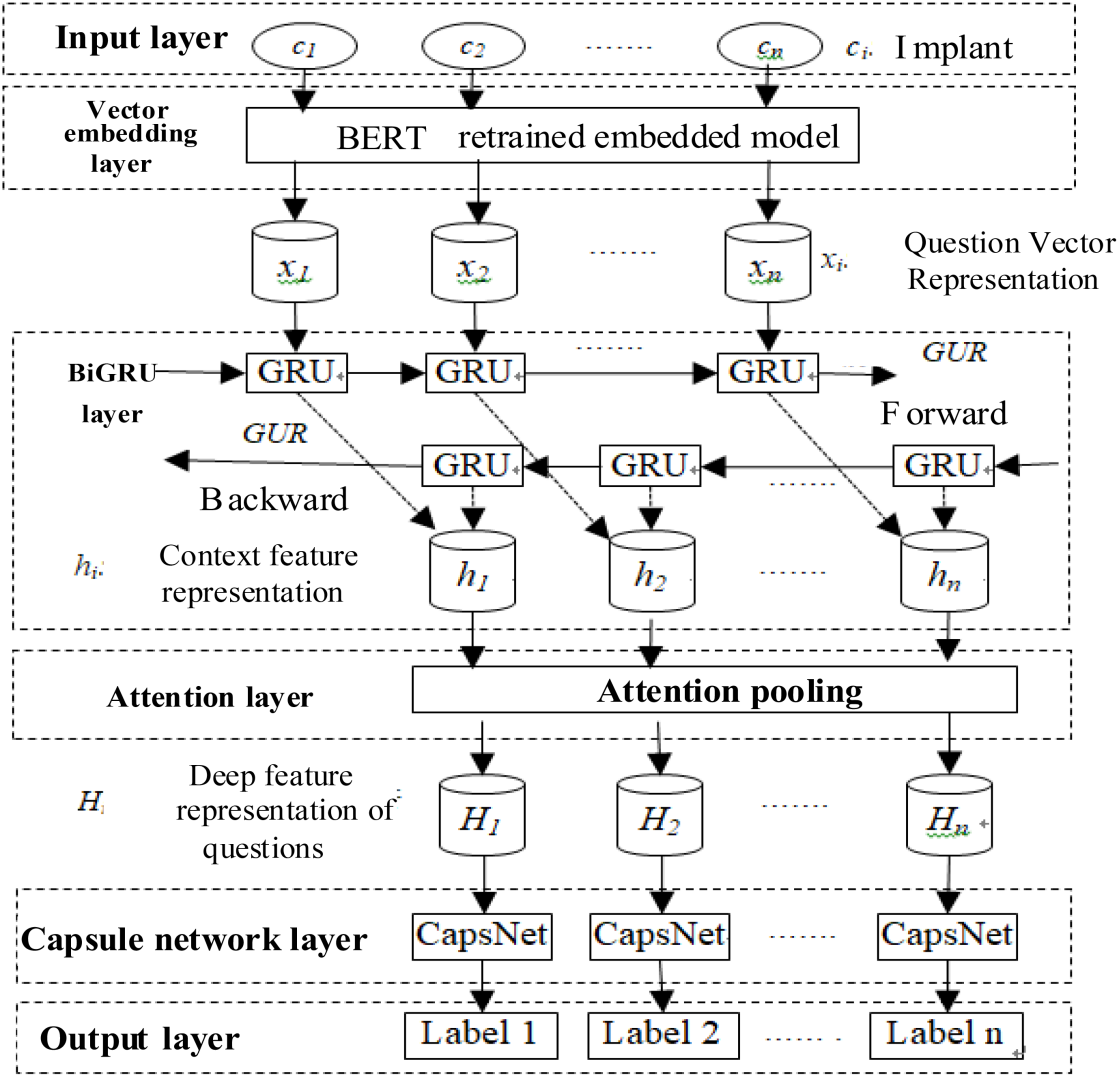
Architecture of BBGCAP, consisting of input and output layers, BERT vector embedding layer, BiGRU feature extraction layer, attention pooling layer, and capsule network layer.

Aiming at the characteristics of small vocabulary, strong sparse features, large noise, and poor normalization in crop disease-pest Q&A questions, a crop disease-pest question classification method, namely, BBGCAP is proposed. Its basic architecture is shown in **Figure 1**.

In the method, question feature vocabulary is extended, word vector is weighted according to the importance of the vocabulary, text features are extracted using BiGRU and CapsNet, and its structure and parameters are further optimized by cross-validation strategy. The main components of BBGCAP are introduced in detail as follows.

### 3.1 Question participle

In question-answer system, each sentence is first segmented. Word segmentation is the addition of boundary markers between words in Chinese sentences. There are many methods for word segmentation, including shortest path word segmentation, N-Gram word segmentation, recurrent neural network (RNN) word segmentation, and transformer word segmentation. There are also many word segmentation tools, such as Jieba, HanLP, and FoolNLTK. Most word segmentation tools, such as Institute of Computing Technology Chinese Lexical Analysis System (ICTCLAS) of the Chinese Academy of Sciences, Language Technology Platform (LTP) of Harbin Institute of Technology, and Jieba, have accuracy rates of more than 95%. This paper uses the Jieba word splitter and adds it to the agricultural domain dictionary, so that domain vocabulary can be correctly segmented, with spaces between words as segmentation, as shown in **Table 1**.

**Table 1:** Crop pest question sentence segmentation.

Question before participle	Question after participle
How to deal with the phenomenon of green and black rice caused by excessive fertilization?	Rice, spread manure, too much, appear, bluish black, phenomenon, how, handle
What are the environmental conditions that affect rice tillering?	Impact, rice, tillering, environment, conditions, what, what
There are a large number of snails in the rice field, how to prevent and control them?	Paddy fields, with a large amount of, and snails, how, prevent and control them
How to control rice blast?	Rice, blast, how, prevention and cure
How to treat bacterial stripe disease of rice?	How, treat, rice, bacterial, stripe disease
What are the characteristics of rice seedling disease?	Rice, cachexia, what, characteristics
What are the most effective prevention and control methods for rice seedling disease?	Rice, rice seedling disease, the most, effective, prevention and control. Methods, have, what
Can raising ducks in rice fields prevent insect pests?	Paddy fields, duck farming, can, prevention, pest infestation
Can fish farming in rice fields prevent and control disease-pests?	Paddy fields, fish farming, can, prevention and control, disease-pests

### 3.2 BERT vector embedding

**Figure 2:**
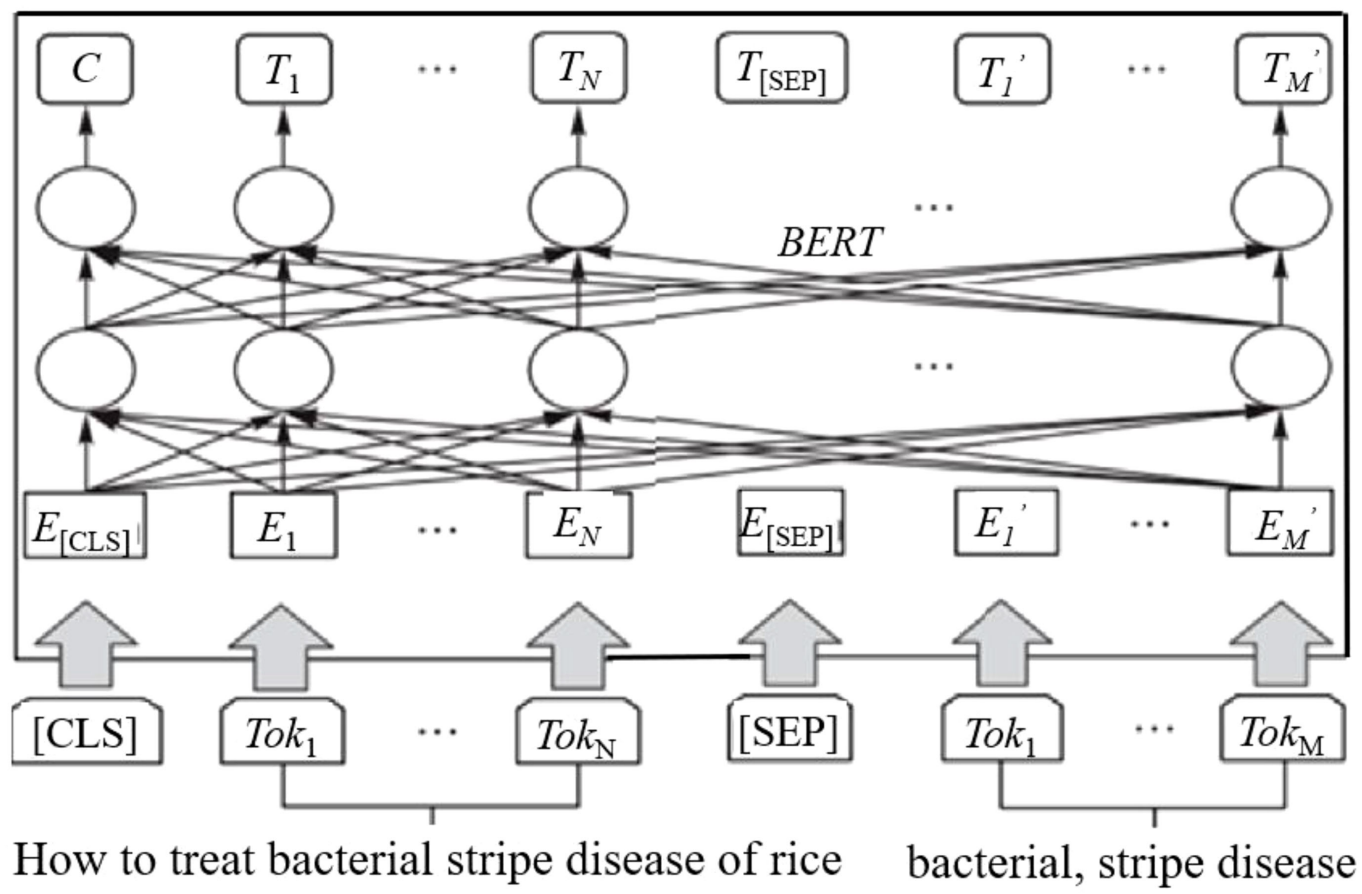
BERT structure, where [CLS] and [SEP] are marked at the beginning and end of the sentence, respectively, and Tok*_i_* is the *i*th token, randomly blocking some characters; E*_i_* is the embedding vector of the *i*th token, and T*_i_* is the feature vector obtained from the *i*th token after BERT processing.

BERT is a pre-trained language model for bidirectional encoding representation of transformers. It generates deep bidirectional language representation, has a deeper understanding of context than Word2vec and unidirectional language models, and can extract more efficient vector features from corpus. After inputting the word segmentation of crop disease-pest question sentences into BERT, it is transmitted to the word embedding layer, including marker word embedding, sentence word embedding, and positional word embedding, as shown in **Figure 2**.

Output the corresponding word vector for each word in the sentence through BERT, where the maximum length of the sentence is set to *L*, and the word vector dimension is *V.* Generate the word vector matrix *X* as follows:


(1) $$X=\left[X_{1},X_{2},X_{3},\ldots ,X_{n}\right]{\text{∈ ℝ}}^{L\times V}$$

### 3.3 BiGRU feature extraction

**Figure 3:**
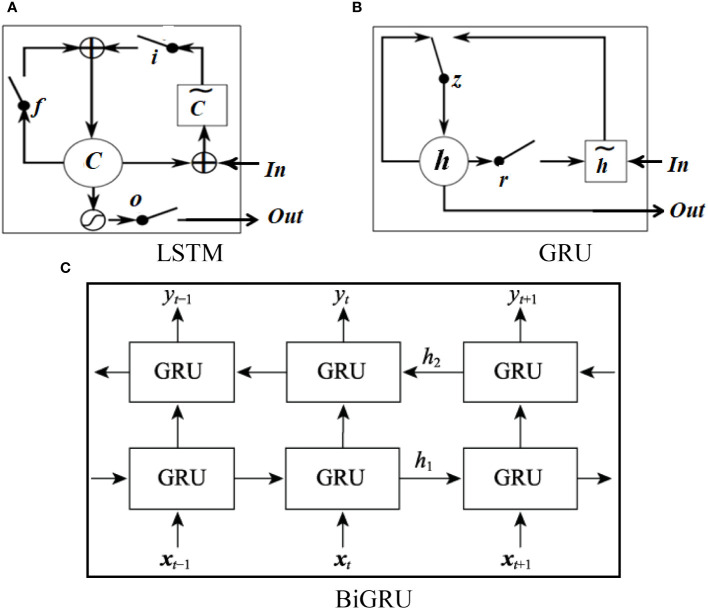
Structure of LSTM, GRU, and BiGRU, where *i*, *f*, and *o* in **(A)** represent input, forgetting, and output gates, respectively; *C* and $\tilde{C}$ represent memory cells and new memory cell contents, respectively; *r* and *z* represent reset and update gates in **(B)**, respectively; and *h* and $\tilde{h}$ are activation and candidate activation gates, respectively. *x_t_* in **(C)** is the input vector at time *t*, *y_t_* is the output vector at time *t*, *h_1_* and *h_2_* are the output of the hidden layer state and update state at time, respectively.

LSTM and GRU are two variants of RNNs that use gating mechanisms to track the sequence state, where GRU is simpler and superior to LSTM when the input data are scarce or the risk of overfitting is high. GRU consists of reset gates and update gates, which selectively pass through information through a “gate” structure, capturing sequence length dependencies and contextual information, thereby solving the problem of gradient vanishing or exploding in recursive networks. Their structures are shown in **Figures 3A, B**.

For time *t*, its GRU state is calculated as follows (Islam et al., 2022; Ma et al., 2022):


(2) $$\begin{array}{c} Z_{t}=\sigma (W_{z}\cdot \left[h_{t-1},x_{t}\right]+b_{z})\\ r_{t}=\sigma (W_{r}\cdot \left[h_{t-1},x_{t}\right]+b_{r})\\ {\tilde{h}}_{t}=\tanh(W_{h}\cdot \left[r_{t}*h_{t-1},x_{t}\right]+b_{h})\\ h_{t}=(1-z_{t})*h_{t-1}+Z_{t}*{\tilde{h}}_{t} \end{array}$$

where $x_{t}$ is the input vector at time *t*; σ is the sigmoid activation function; $W_{z},W_{r},$ and *W_h_* are the weights; $b_{z},b_{r},$ and *b_h_* are bias; *Z_t_* and *r_t_* are the current unit state of the control gate and update gate at time *t*, respectively; $h_{t}$ and ${\tilde{h}}_{t}$ are the output of the hidden layer state and update state at time *t*, respectively.

GRU ignores future contextual information, where BiGRU can train a GRU model forward and backward using the same training sequence and then linearly combine the outputs of the two models to ensure that each node in the sequence can fully rely on all contextual information. Therefore, for question classification tasks, BiGRU is often used to better understand the user intentions. Its structure is shown in **Figure 3C**. For a given *i*th participle, BERT embeds the word *E*(word_i_), and its output at time *t* is calculated as follows:


(3) $$h_{it}=\left[{\vec{h}}_{it},{\overset{\leftarrow }{h}}_{it}\right]$$

where ${\vec{h}}_{it}=GRU(E(word_{i}),{\vec{h}}_{it-1})$ and ${\overset{\leftarrow }{h}}_{it}=GRU(E(word_{i}),{\overset{\leftarrow }{h}}_{it-1})$ are the outputs of forward GRU and backward GRU at time *t*, respectively.

### 3.4 Attention pooling

**Figure 4:**
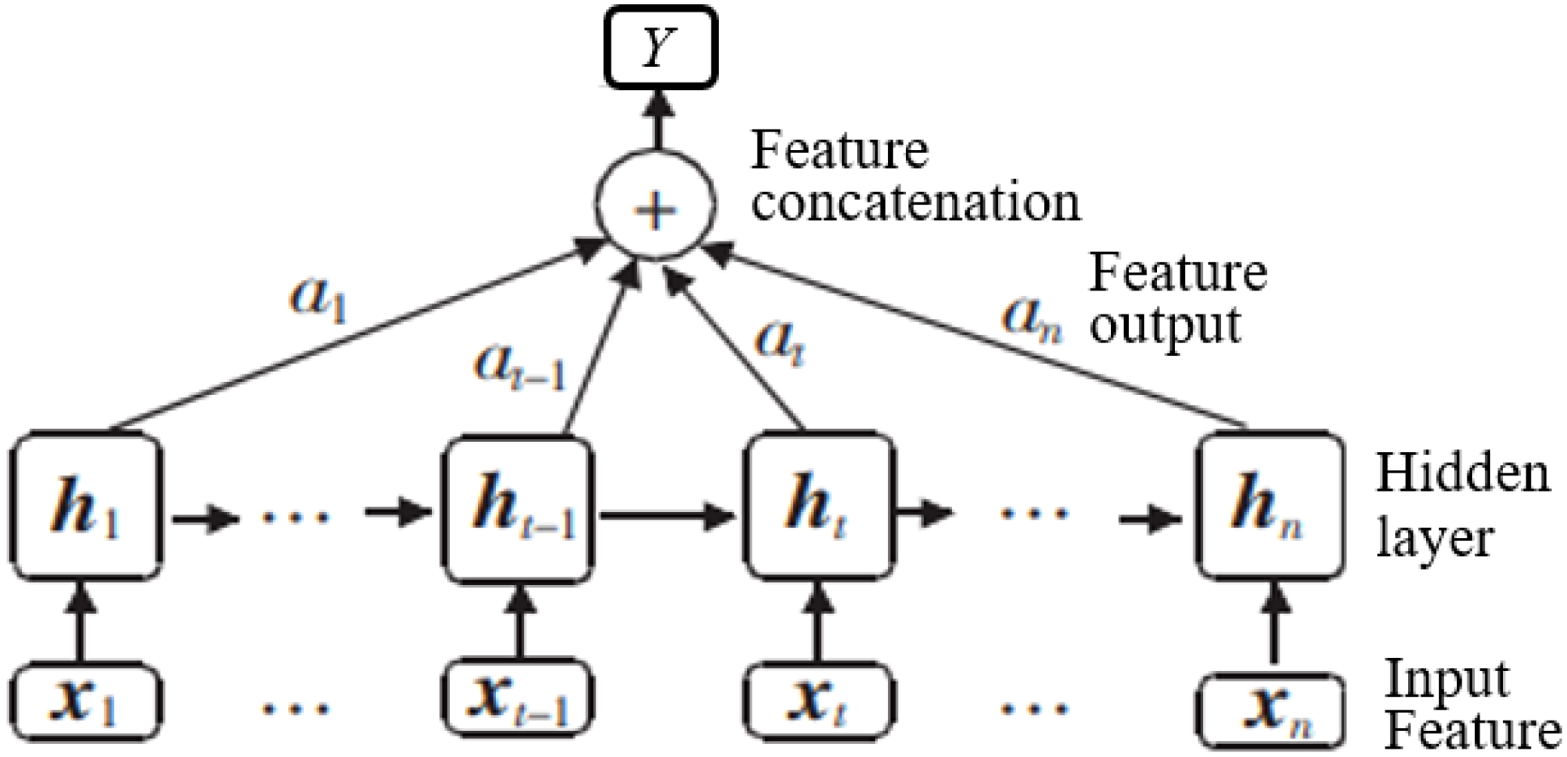
Structure of attention mechanism, where *x_i_* is the *i*th input and *α_i_* is the *i*th weight coefficient of the state *h_i_.*.

Attention mechanism is often embedded in machine learning and natural language processing models, such as YoloV3, U-Net, BERT, GPT, and transformer. It aims to allow the model to focus on the most crucial feature information relevant to the current task, thereby reducing attention to other irrelevant or noisy information. It can automatically learn and calculate the contribution of the input data to the output data. Its structure is shown in **Figure 4**.

To highlight the importance of different words in the entire question-answer classification, BiGRU introduces an attention layer. Its input is the output vector *h_it_* activated by BiGRU in the previous layer, and the attention score *a_it_* is calculated as follows:


(4) $$\begin{array}{c} S_{it}=\sum_{i-1}^{T}a_{it}h_{it}\\ u_{it}=\tanh(w_{w}h_{it}+b_{w})\\ a_{it}=\exp(u_{it}^{T}u_{w})/\sum_{i=1}\exp(u_{it}^{T}u_{w}) \end{array}$$

where *h_it_* is the output vector of the previous layer of BiGRU, *w_w_* is the weight coefficient, and *b_w_* is the bias coefficient.

### 3.5 Capsule network

CapsNet consists of three layers: convolutional layer, main capsule layer using vectorized capsules, and convolutional capsule layer using dynamic routing mechanism. Let this layer contain *N_1_* convolution kernel $W\in R^{K\times V}$ , and the word vector element *y_i_* is convoluted as follows:


(5) $$y_{i}=f(W⊗x_{i:i+K-1}+b)$$

where *f* is a ReLU activation function and *b* represents its bias.

Calculate the feature matrix *Y* as follows:


(6) $$Y=\left[Y_{1},Y_{2},Y_{3},\cdots ,Y_{N_{1}}\right]\in R^{(L-K+1)\times N_{1}}$$

The main capsule layer is different from CNNs. This layer integrates semantic features of the same position in sentences, saves them as vectorized capsules, and converts the feature matrix obtained in the previous step into a capsule matrix *Z* through *N_2_ m-*dimensional transformation matrices $N_{2}\times 1\times m$ :


(7) $$Z=\left[Z_{1},Z_{2},Z_{3},\cdots ,Z_{N_{2}}\right]\in R^{(L-K+1)\times N_{2}\times m}$$

Perform a linear transformation on the *K_1_*-row capsules of *Z* through *N_3_* transformation matrices of *m × m*, transformation matrix of *N_3_*, and calculate the prediction vector $z_{i}$ as follows:


(8) $$z_{i}=W_{3}z_{i}+b_{i}$$

Weighted sum operation on $z_{i}$ yields *u_j_*


(9) $$u_{j}=g(\sum_{i}c_{i}z_{i})$$

where *c_i_* is the coupling coefficient updated during the dynamic routing process, which is obtained by calculating *b_i_* of the connection between capsule $z_{i}$ in this layer and capsule *u_j_* in the upper layer through the softmax function. The update method for *b_i_* is


(10) $$b_{i}\leftarrow {b^{\prime }}_{i}+u_{j}\cdot z_{i}$$

where ${b^{\prime }}_{i}$ is the weight obtained from the previous iteration, initialized to 0.

Calculate the capsule matrix *U* as follows:


(11) $$U=\left[U_{1},U_{2},\cdots ,U_{N_{3}}\right]\in R^{(L-K-K_{1}+2)\times N_{3}\times m}$$

Finally, softmax is adopted as the feature classifier. Softmax normalizes the output feature vector and maps it to the (0, 1) interval to obtain the probability values of the corresponding output features for each type of question, thereby classifying the question.

The gradient descent–based method is adopted to learn the parameters of BBiQLSTMA. In each training time, for *L* input samples ⟨*x*_i_, *y*_i_⟩, the gradient of each parameter relative to the model loss is calculated and then updated each parameter with learning rate λ:


(12) $$\begin{array}{c} Loss=\sum_{i=1}^{L}-\log p(y_{i}|x_{i})\\ \theta =\theta -\lambda \frac{\partial Loss}{\theta } \end{array}$$

where *θ* is the super parameter and λ is learning rate.

**Figure 5:**
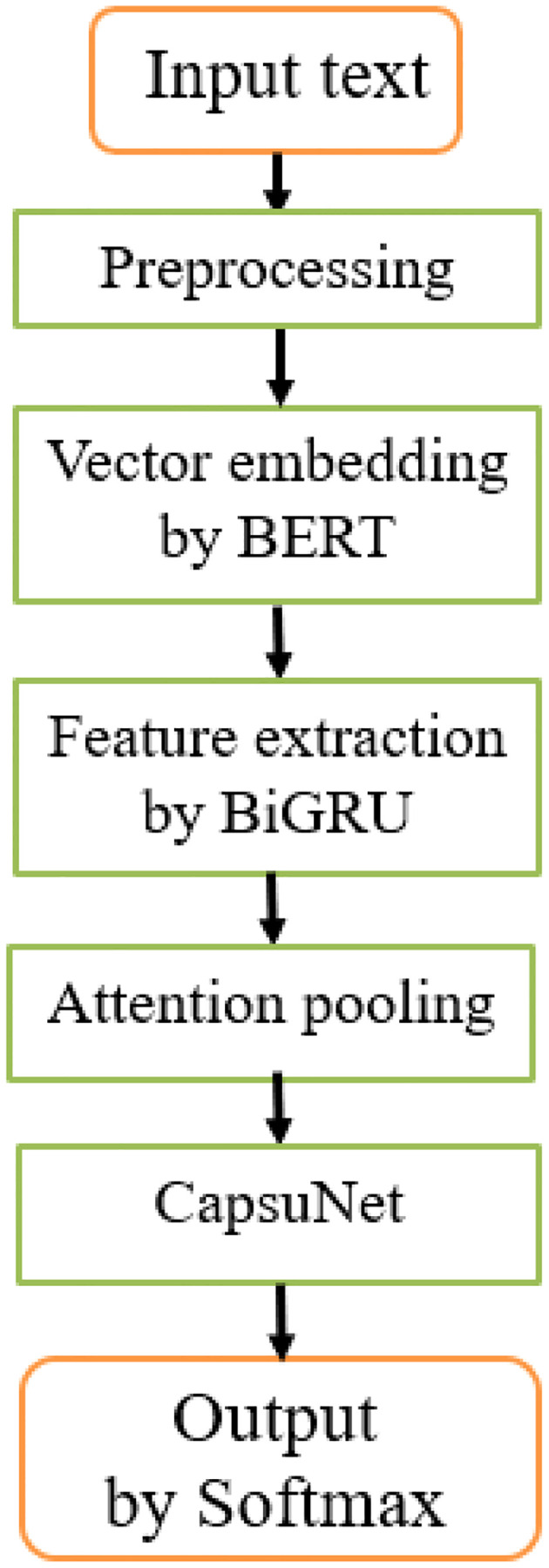
The flowchart of the methodology.

From the above analysis, a BBGCAP-based crop disease-pest knowledge question classification method is proposed. Its flowchart is shown in **Figure 5**.

The pseudocode of the algorithm is given as follows:

Input crop disease-pest question text *T*: mini-batch T :


$$T=T_{1},\cdots ,T_{m}$$

Output:The label of crop disease-pest question text *T.*

1. $X=\left[X_{1},X_{2},X_{3},\ldots ,X_{n}\right]{\text{∈ ℝ}}^{L\times V}$ , the corresponding word vector for each word in the sentence through BERT( T ), *T* is a text.

2. $h_{it}=\left[{\vec{h}}_{it},{\overset{\leftarrow }{h}}_{it}\right]$ ,   ${\vec{h}}_{it}=GRU(E(word_{i}),{\vec{h}}_{it-1})$ and ${\overset{\leftarrow }{h}}_{it}=GRU(E(word_{i}),{\overset{\leftarrow }{h}}_{it-1})$ are the outputs of forward GRU and backward GRU at time *t*, respectively.

3. $S_{it}=\sum_{i=1}^{T}a_{it}h_{it}$ , $u_{it}=\tanh(w_{w}h_{it}+b_{w})$ , $a_{it}=\exp(u_{it}^{T}u_{w})/\sum_{i=1}\exp(u_{it}^{T}u_{w})$ , where *h_it_* is the output vector of the previous layer of BiGRU, *w_w_* is the weight coefficient, and *b_w_* is the bias coefficient.

4. Calculate the prediction vector $z_{i},z_{i}=W_{3}z_{i}+b_{i}$ .

5. Weighted sum operation on $z_{i}$ yields *u_j_*, $u_{j}=g(\sum_{i}c_{i}z_{i})$ .

6. Update method for *b_i_*, $b_{i}\leftarrow {b^{\prime }}_{i}+u_{j}\cdot z_{i}$ .

7. Calculate the capsule matrix *U*, $U=\left[U_{1},U_{2},\cdots ,U_{N_{3}}\right]\in R^{(L-K-K_{1}+2)\times N_{3}\times m}$ .

8. Label (*T*) = Softmax(*U*), it maps the output of multiple neurons to the interval (0, 1), which can be understood as a probability, so as to carry out multi-classification.

9. Output the label of *T.*

## 4 Experimental results and analysis

BBGCAP is verified on the constructed crop disease-pest question dataset and compared with three crop disease-pest knowledge question classification methods: Chinese agricultural disease-pests named entity recognition with multi-scale local context features and self-attention mechanism (MSLCFSA) (Guo et al., 2020), question classification method based on merge-convolutional neural networks-deep pyramid convolutional neural networks–long short-term memory (MCDPLSTM) (Yu et al., 2021), and text classification model based on CNN and BiGRU fusion attention mechanism (CNNBiGRUA) (Ma et al., 2022). Among them, Word2vec is used for word embedding, whereas BBGCAP employs BERT for word embedding. The model parameters are initialized using Xavier normal distribution. The experimental conditions are set as follows: the hidden state dimension of GRU unit is set to 100; the output vector dimension of BiGRU is also set to 100; the iteration number of CapsNet is set to 10 (default is 5); the embedding size is 128; the hidden size is 768; the batch size is 32; the original learning rate is 0.001; the number of iterations is 3,000; the hidden activation is ReLU; the attenuation rate is 0.1, the hidden layer attenuation rate is 0.5; and other weights, biases, and other parameters change continuously with model optimization. BiGRU and CapsNet are conducted on Keras, TensorFlow1.7.0, and PyTorch library frameworks, whereas Direct Data Ingestion (DDI) extraction experiments are conducted on Ubuntu 18.04LTS as the operating system, 32GB of memory, Intel Core i5-4200U CPU @ 2.30 GHz, GPU GEFORCE GTX 1080ti, and Ubuntu 14.0. BERT, BiGRU, and CapsNet are optimized by Adam Optimizer. Evaluate its performance using precision, recall, and F1 and calculate as follows:


(13) $$Precision=\frac{TP}{TP+FP},Recall=\frac{TP}{TP+FN},F1=\frac{2\cdot Precicion\cdot Recall}{Precicion+Recall}$$

where *TP* (true positive) is the number of correctly classified positive instances, *FP* (false positive) is the number of misclassified positive instances, and *FN* (false negative) is the number of misclassified negative instances.

### 4.1 Dataset

Through Scrapy crawler framework, various Chinese text corpora of common crop disease-pests are captured on various Baidu Encyclopedia, Interactive Encyclopedia, Chinese Wikipedia, as well as crop management websites such as “Expert Online System,” “Planting Q&A Network,” and “Nanjing Agricultural Commission,” including various questions and sentences from agricultural producers about common crop disease-pests. Some crop disease-pest information processing methods are adopted to correct the crawled corpus, remove duplicate and unclear data, and select 3,000 clear question-answer pairs as the dataset of frequently asked questions and corresponding answers. The questions are converted into narrative sentences to construct a dataset of common crop disease questions. Some questions and their classification are shown in **Table 2**.

**Table 2:** Examples of crop pest questions.

Questions about crop disease-pests	Category
What are the common disease-pests of apple trees? How to prevent and treat it?	Apple disease-pests
Apple ring rot mainly harms branches and fruits, how to prevent and control it?	Apple Ring Rot
What disease is the initial appearance of light brown small round spots on the surface of apples, which have expanded into deep brown?	Apple anthracnose
Apple rot is a destructive disease that occurs on the fruiting trunk and branches. How to prevent and control it?	Apple canker
How to prevent and control common diseases in apples caused by the infection of mountain rust fungus?	Apple rust
How to prevent and control apple diseases that are prone to frequent rainfall and high temperatures?	Apple rust
Rust mainly affects the leaves of apple plants and can also harm the tender parts of the plants. How to prevent and control it?	Apple rust
What disease is apple leaves that seem to rust? How can they be prevented and treated?	Apple rust
Watermelon skin has various waterlogged lesions, some of which crack and rot. What is this disease?	Watermelon fruit rot disease
What is the disease of irregular brown lesions appearing on the midrib of watermelon leaves, which extend to the leaf edge and appear waterlogged?	Watermelon fruit rot disease
How to prevent irregular or circular red brown spots on grape leaves?	Grape rotation disease
There are parallel opaque gray black spots on corn leaves. How to prevent them?	Gray leaf spot
What is the disease of corn leaves turning yellow and withering, with gray black mold layers appearing on both sides of the leaves in the later stage of disease spots?	Gray leaf spot
What is the disease on the stem and fruit, with elliptical, brown, slightly concave spots and scattered small black spots on the spots?	Tomato powdery mildew
Tomatoes are prone to disease under high temperature and humidity during drought, and white powder appears on their leaves. What is this disease?	Tomato spot blight
What is the disease of tomato leaves with powdery spots and circular powdery spots on the front and white powdery substance on the surface?	Tomato powdery mildew
What is the disease of tomato with elliptical brown spots on the petioles and stems, and black small dots on them?	Tomato spot blight
How to prevent tomato cracking under high temperature, strong light, and drought?	Tomato diseases
What are the symptoms of tomato virus disease?	Tomato diseases
What is the reason for tomato leaf turning yellow? How to prevent and treat it?	Tomato diseases
Black mold appears on carrot spots, how to prevent and control it?	Carrot black spot disease
What is the disease of citrus yellow shoot peripheral branches or tree top leaves that are prone to yellowing and yellowing.	Citrus huanglongbing
When the humidity is high, the peanut leaves have a gray brown powdery mold layer, and the disease spots are oval shaped and dark brown. What is this disease?	Peanut brown spot disease
Eggplant can develop diseases in all parts of the plant from the seedling stage to the mature stage, and the fruit is the most severely affected. What is this disease?	Eggplant phomopsis rot
What disease is the appearance of circular yellow spots on the leaves of apple trees with red edges?	Apple rust
What is the disease of irregular shaped lesions on the reddish brown leaves and a gray brown mold layer on the back when moist?	Grape ringspot disease
What are small brown spots on carrots with yellow circles on the edges?	Carrot black spot disease
The veins and surrounding tissues of citrus leaves turn green. What is yellowing of the mesophyll?	Citrus huanglongbing
What are the elliptical brown spots on the petioles and stems of peanuts?	Peanut brown spot disease
What are the small black spots with brown spots and wheel patterns on eggplants?	Eggplant phomopsis rot
Can the little ground tiger and the cotton bollworm use the same medicine?	Cotton pest damage
How to prevent and control adult and nymphs from consuming leaves or tender head juice?	Cotton pest damage

### 4.2 Results

**Figure 6:**
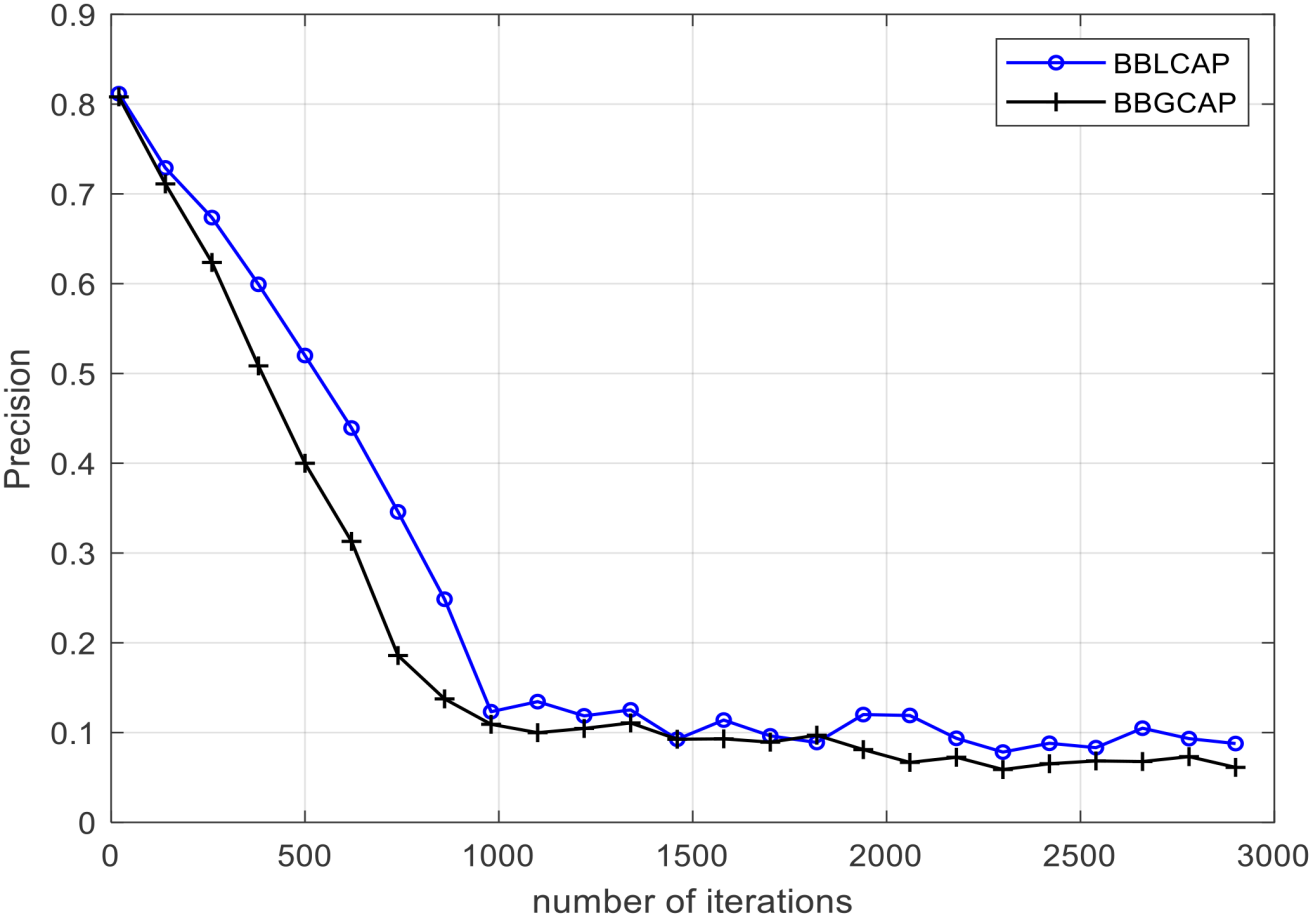
The precision versus iteration.

In the experiments, the word vector dimension is set to 128, the maximum length of the question is set to 100, and each GRU output feature dimension in the BiGRU layer is set to 128. Stacking mode is selected to connect the outputs of forward and backward GRUs. The experiments are conducted using a 10-fold cross-validation method, i.e., conducting 10 experiments. During each experiment, 10% of the questions in each category are randomly selected as the test dataset, and the remaining data are used as the training dataset. The test dataset and training dataset do not overlap. The average test results of the 10 test datasets are used as an evaluation indicator for the model classification performance. In the data preprocessing process, impurity questions are removed, and the Jieba word segmentation tool is used to segment user questions, removing stop words, punctuation marks, and special characters. Then, BERT is provided by Google open-source is used for training to quantify questions. CapsNet first extracts a set of features, then makes a cluster on this set of features, predicts with the clustering results, carries out backpropagation according to the predicted results, updates the matrix of extracted features that can be understood as changing a set of features or fine-tuning the features, and continues to cluster, and the cycle repeats. Finally, softmax is used to classify questions. The precisions versus iteration of BBGCAP and BBLCAP are shown in **Figure 6**, where BBLCAP is a variation of BBGCAP with BiLSTM replacing BiGRU, and the rest remaining unchanged. From **Figure 6**, it is seen that BBGCAP and BBLCAP converge at 2,500 iterations, and the convergence performance of BBGCAP is better than that of BBLCAP. In the following, the number of iterations is set as 3,000.

The overall classification performances of BBGCAP and three comparative models on the test set are shown in **Table 3**.

**Table 3:** Word segmentation classification of crop pest questions.

Result Method	Precision	Recall	F1
MSLCFSA	88.73	85.43	87.05
MCDPLSTM	83.56	80.92	82.22
CNNBiGRUA	86.49	81.04	83.68
BBGCAP	92.15	93.17	92.66

From **Table 3**, it can be seen that the proposed model in this article is superior to the other three methods. The main reason is that the proposed model BBGCAP outperforms other models because it fully utilizes the advantages of three components: BERT is superior to Word2vec, BiGRU is superior to BiLST, and attention pooling is better than attention mechanism in MSLCFSA and CNNBiGRUA. MSLCFSA is superior to CNNBiGRUA and MCDPLSTM because it can obtain the multi-scale local context features and utilizes attention mechanism to fully reflect the keyword features in questions, making the question classification model have better accuracy in question feature extraction, thereby improving classification accuracy. Hybrid model CNNBiGRUA is little better than MCDPLSTM because it utilizes the advantages of CNN, BiGRU and attention mechanism, where BIGRU fully uses the positional information before and after sentence segmentation.

To test the impact of the number of training samples on the question classification results, different fold cross-validation experiments are carried on and the results are shown in **Table 4**.

**Table 4:** Results of BBGCAP with different fold cross-validation experiments.

Fold cross-validation Result	Four-fold	Six-fold	Eight- fold	Nine-fold
Precision	71.16	78.11	87.26	91.14
Recall	72.05	79.34	87.82	90.47
F1	71.60	78.72	87.54	90.80

From **Table 4**, it is seen that the number of training samples has a significant impact on the effectiveness of the model, mainly due to the variety of user questions and the lack of a fixed format, requiring a large number of samples for training. As the dataset increases, the classification results significantly increase.

To test the advantages of BERT, BiGRU, and attention pooling mechanisms, we improve the structure of the proposed model BBGCAP and conduct 10-fold cross-validation experiments under the unchanged experimental conditions. The results are given in **Table 5**.

**Table 5:** Results of ablation experiments.

Change structure Result	Word2vec replaces BERT	BiLSTM replaces BiGRU	Delete attention pooling	CNN replaces CapsNet
Precision	86.20	88.51	83.62	81.71
Recall	85.24	84.43	86.11	82.66
F1	85.72	86.42	84.85	82.18

From **Tables 3**–**5**, it can be seen that the effectiveness of crop disease-pest question classification not only depends on the selection of question classification algorithms but also has a significant impact on the size of the model training dataset. The experimental results in **Tables 3**–**5** validate that BBGCAP is effective and feasible for crop disease-pest intelligent question answering system, which is a real-time and practical system and requires high accuracy. As a key step of problem classification, when training the selected model, the training data should contain as many problems as possible to improve the accuracy of the whole system.

## 5 Conclusion

The crop disease-pest–related question classification is an important and challenging problem in the crop disease-pest question answering system. A BERT-BiGRU-CapsNet with attention pooling model, namely, BBGCAP, is constructed for the crop disease-pest Q&A system. BBGCAP is a hybrid network model, integrating the advantages of BERT, BiGRU, CapsNet, and attention pooling. The experimental results demonstrate that BBGCAP outperforms the other methods. In fact, BBGCAP has hierarchical structures and requires a lot of optimization in sample training. The future work is to optimize BBGCAP and aims to address the complex and diverse semantic information of crop disease-pest user-questions.

## Data availability statement

The raw data supporting the conclusions of this article will be made available by the authors, without undue reservation.

## Author contributions

TZ: Methodology, Writing – original draft. DW: Resources, Validation, Writing – review & editing.
